# Microbial population dynamics under microdoses of the essential oil arborvitae

**DOI:** 10.1186/s12906-019-2666-6

**Published:** 2019-09-05

**Authors:** Rhegan C. McGregor, Kory A. Parker, Jacob M. Hornby, Leigh C. Latta

**Affiliations:** Division of Natural Sciences and Mathematics, Lewis-Clark State College, 500 8th Avenue, Lewiston, ID 83501 USA

**Keywords:** *Staphylococcus aureus*, *Pseudomonas aeruginosa*, *Escherichia coli*, *Candida albicans*, *Candida auris*, Arborvitae essential oil, Carrying capacity (*K*), Intrinsic rate of growth (*r*), Hydrophilic

## Abstract

**Background:**

With the current concern caused by drug resistant microorganisms, alternatives to traditional antimicrobials are increasingly necessary. Historical holistic treatments involving natural approaches are now of interest as a potential alternative. Many essential oils have antimicrobial properties with the ability to modify bacterial and fungal population dynamics in low concentrations.

**Methods:**

In this study, bacterial and fungal growth in response to varying concentrations of arborvitae oil was assessed using spectrophotometric methods to obtain estimates of population growth parameters including carrying capacity (*K*) and intrinsic rate of growth (*r*). Estimates of these parameters were compared among doses within strains using general linear modeling.

**Results:**

Results suggest the active component of the essential oil arborvitae is likely of hydrophilic nature and demonstrates the ability to influence both *K* and *r* during bacterial and fungal growth in a dose-dependent manner. Highly concentrated doses of arborvitae completely kill *Escherichia coli* and significantly inhibit *Staphylococcus aureus*, however these same doses have no effect on *Pseudomonas aeruginosa*. Accordingly, microdoses of arborvitae demonstrated the ability to inhibit population growth parameters in both prokaryotic and eukaryotic microorganisms. Specifically, *K* of *E. coli*, *r* of *Candida auris*, and both *K* and *r* of *Candida albicans* were significantly reduced in the presence of microdoses of arborvitae.

**Conclusions:**

Microdoses of essential oils have the ability to inhibit one or both population parameters in both prokaryotic and eukaryotic microorganisms. Some microorganisms appear to be more susceptible to this essential oil arborvitae than other microorganisms. The use of essential oils, such as arborvitae, as novel antimicrobials may prove useful when contending with the current epidemic of multidrug resistant pathogens.

**Electronic supplementary material:**

The online version of this article (10.1186/s12906-019-2666-6) contains supplementary material, which is available to authorized users.

## Background

Rapidly evolving microbes display high resistance to various antimicrobial drugs and present both pressing and complicated challenges for the management of human health.

Due to high costs and investments, and traditionally low yields, many pharmaceutical companies have abandoned development of novel antimicrobials in favor of a focus on drugs used chronically that potentially impart desirable benefits [1]. Advances in medicine, coupled with the misuse and overuse of antimicrobials, greatly contribute to the evolution of multi-drug resistant pathogenic microbes. Multi-drug resistance can evolve as a consequence of the interaction between mutation and natural selection, or mutation and genetic drift. Physiological environments in which antimicrobial drugs are prevalent at lethal concentrations result in strong selection for resistant microbes [2, 3]. One current hypothesis that may explain these results is that lethal antibiotic levels select for beneficial mutations that confer resistance and increase fitness in microbial populations [3, 4]. Genetic drift can also result in the evolution of drug resistance, particularly during exposure to microdoses of antimicrobial drugs, because the drugs increase the rate of genome-wide mutation in microbial strains [5]. As a consequence of this elevated mutation rate, the number of new beneficial mutations that give rise to resistance to these antimicrobial drugs also increase in frequency. Although the vast majority of new mutations are deleterious, resistance can evolve with only one or a few beneficial mutations [6].

Prior to modern medicine, natural remedies and herbal supplements were used to promote and maintain human health. Historical records dating back to 4500 BC document that ancient Egyptians used various essential oils extracted from plants such as myrrh and cedar, in addition to onions and grapes, for topical ointments, perfume fragrances, cosmetics, and medications [7]. One use for arborvitae oil that is once again gaining popularity is as an additive in skincare products [8]. As potential alternatives to prescribed antimicrobial drugs, these early medical practices that leveraged natural remedies, such as essential oils, are currently under scientific investigation to assess their effectiveness as antimicrobial treatments. Cinnamon oil extract inhibits growth in multiple species of fungus [9] and various species of Gram-negative and Gram-positive bacteria [10]. Other essential oils, such as ajwain and ginger, are known inhibitors of multi-drug resistant *S. aureus* when supplied in aliquots of less than 11 μL, and induce inhibition zones greater than the prescribed antibiotics vancomycin and linezolid [11]. Although many essential oils display some degree of antimicrobial properties, the underlying mechanisms by which the oils inhibit microbial growth are not clearly understood. The leading hypothesis that explains the mechanisms of oil-induced antimicrobial activity revolves around the chemical composition of the oil, in particular the functional groups [12]. Specifically, 1,8-cineole and α-terpineol are two chemical components found in multiple essential oils with confirmed antimicrobial properties that cause cell wall and cell membrane lysis in both Gram-positive and Gram-negative bacterial species [13].

This study investigates the antimicrobial properties of the essential oil *Thuja plicata* in both Gram-positive and Gram-negative bacterial species, and two species of fungi. Related to *Thuja plicata, Thuja orientalis*, which is often referred to as the American arborvitae and is native to eastern North America, has been shown as an essential oil to have antimicrobial activity against multiple bacterial and fungal organisms [14]. *Thuja plicata*, the giant arborvitae tree, is native to the Pacific Northwest Region belonging to the cypress family and is known for its unique resistance to decomposition and harmful pests [15–17]. The dynamics of population growth were assessed in terms of carrying capacity (*K*) and intrinsic rate of growth (*r*) for each species under variable doses of arborvitae in order to evaluate the potential antimicrobial properties of arborvitae essential oil. In the context of this experiment, estimates of *K* describe the maximum cell density and reflect the population size at which rates of cell division are equal to rates of cell death. Estimates of *r* describe the maximum rate of increase in cell density during the exponential growth phase and reflect the difference between the increased rate of cell division relative to rates of cell death.

Results from this study provide insight into the anti-microbial properties of the essential oil arborvitae across both prokaryotic and eukaryotic microorganisms. Specifically, dose-response analysis of estimates of *K* and *r* indicate increasing microdoses of arborvitae essential oil decrease *K*, but have no effect on *r*, for the Gram-negative species *Escherichia coli*. However, the essential oil arborvitae increased *r*, but had no effect on *K*, in the Gram-positive species *Staphylococcus aureus.* Arborvitae had no effect on either *K* or *r* for the Gram-negative species *Pseudomonas aeruginosa.* The essential oil arborvitae had the greatest impact on both population parameters in the fungal species *Candida albicans* as both *K* and *r* decreased as arborvitae dose increased. Interestingly, *r* for the fungal species *Candida auris* decreased in the presence of arborvitae oil, but *K* did not change in the presence of arborvitae oil. Concisely, arborvitae essential oil alters the population dynamics of both prokaryotic and eukaryotic organisms. These findings suggest there are chemical components of arborvitae oil with antimicrobial properties, and more generally that essential oils may provide an alternative treatment to prescribed antimicrobial drugs.

## Methods

### Species used

Three standard bacterial laboratory strains (*Staphylococcus aureus, Escherichia coli, and Pseudomonas aeruginosa)* in addition to two fungal species (*C. albicans* (strain SC5314) and *C. auris* (CAU-07, CDC and FDA Antibiotic Resistance Isolate Bank. Atlanta (GA): CDC. [obtained Jan. 2017])) were selected to test the effectiveness of arborvitae oil as a population growth inhibitor in both prokaryotes and eukaryotes. The Gram-positive and Gram-negative prokaryotes vary in the composition of the cell wall, which may influence the effectiveness of the oil. Specifically, the Gram-negative species, *E. coli* and *P. aeruginosa*, have two cell membranes on either side of the thin peptidoglycan layer, whereas the Gram-positive species, *S. aureus*, has a thick peptidoglycan layer that is exterior to the single cell membrane present. Of the fungal species, *C. auris* was of particular interest because of its recent notoriety in the popular press and its resistance to multiple antifungal drugs. In contrast, the fungi *C. albicans* is not highly resistant to antifungals (however has routinely been shown to be one of the more highly invasive and disseminating strains of *C. albicans*) and was chosen as a reference species to compare with *C. auris*.

### Arborvitae essential oil

Essential oil obtained through steam distillation from *Thuja plicata*, the arborvitae tree, was purchased from Doterra®. Arborvitae oil is of interest because the plant has an inherent resistance to microbial decomposition and harmful plant pests. In order to test the effectiveness of arborvitae essential oil as an inhibitor of population growth parameters across microorganisms, a solution was created in a microcentrifuge tube with an aliquot of arborvitae oil suspended in Mueller-Hinton broth for bacteria, or in Glucose Phosphate Proline [18] broth for fungi (Additional file 3: Table S1). This mixture was then vortexed, followed by centrifugation for 5 min at 5000 rpm. The aqueous layer of the biphasic mixture was then removed and added in specific volumes to specified volumes of Mueller-Hinton or GPP broth using a micropipette. Pilot experiments indicated at least one of the active components of the essential oil is hydrophilic (See Additional file 2: Figure S1), therefore this procedure ensures the active component(s) will diffuse into the selected broth.

### Hydrophilicity & High Concentrated Doses of arborvitae oil

In efforts for devising effective methods, the hydrophilic/hydrophobic nature of the essential oil arborvitae was tested. Two solutions with arborvitae oil were created, hydrophilic and hydrophobic, which were then tested against normal growth of *E. coli* in MH broth. Population growth parameters *K* and *r* were compared among the test solutions: hydrophilic, hydrophobic, and MH broth. Arborvitae oil (50 μL) was added directly to Muller-Hinton broth (1 mL) in two separate microcentrifuge tubes. Both tubes were vortexed, but only one tube underwent centrifugation for 5 min at 10,000 rpms. In the centrifuged tube, only the aqueous portion (1 mL) was extracted and added to a larger volume of MH broth (4 mL) to create a hydrophilic solution. In the tube that was only vortexed, 1 mL of the mixed solution was extracted and combined into a volume (4 mL) of MH broth to create the hydrophobic solution. A 96-well plate was loaded and placed into a TECAN Infinite M200 Pro plate reader to measure the absorbance at 600 nm every 15 min for a total of 18.75 h (75 cycles). Samples were maintained at 37 °C and continuously shaken at an amplitude of 5 mm (See Population dynamics Section in Methods).

Highly concentrated arborvitae doses were also prepared to test the bactericidal or bacteriostatic activity of arborvitae oil for each of the bacterial samples: *E. coli*, *S. aureus*, and *P. aeruginosa.* The highly concentrated dose was created with a two-step dilution. First, in a microcentrifuge tube, 50 μL of arborvitae oil was added to 1000 μL of Mueller-Hinton broth. This solution was vortexed and then centrifuged for 5 min as 10,000 rpm (Additional file 3: Table S1 plate M) before the entire 1000 μL aqueous layer of the biphasic mixture of the first diluted solution was then added to 4 mL of Mueller-Hinton broth, creating a final .95% concentrated solution. For the lower concentrated dose (.04%), 2 μL of arborvitae oil was added directly to 4.998 mL of MH broth. As described below in the population dynamics section, liquid inoculations and 96-well plate loading were completed using identical methods (see Population Dynamics Section in Methods). After the 96-well plate was loaded, the plate was placed in the TECAN plate reader to measure the absorbance at 600 nm every 15 min for a total of 18.75 h (75 cycles). The samples were maintained at 37 °C and continuously shaken at an amplitude of 5 mm. A total of two plates were prepared and measured: one contained *E. coli* and the second contained both *S. aureus* and *P. aeruginosa*.

### Population dynamics

A single colony of the desired species grown on Mueller-Hinton agar plates for bacterial species, or YPD agar plates for fungal species, was used to inoculate and propagate liquid cultures in Mueller-Hinton (bacterial) or GPP (fungal) broth. The liquid cultures were shaken at 200 rpm and incubated at 37 °C for 20 h (for bacteria) or 24 h (for fungi). Small aliquots (2 μL) of the desired liquid inoculations were transferred into the designated wells of a 96-well microplate. A two-step dilution process was done to attain the desired doses of arborvitae solutions. The first solution was created with the addition of the arborvitae oil added directly to MH broth (See Solution Prep in Additional file 3: Table S1) before this solution was vortexed and centrifuged at 10,000 rpm for 5 min. The oil slick was removed and the second diluted solution was made with the specified volumes of this solution added to separate tubes with additional MH broth in order to create the final desired concentrated arborvitae doses (Additional file 3: Table S1). Additionally, a control was made with no arborvitae present. Each of the 96 wells contained 200 μL of a specific dose solution in addition to the 2 μL of bacteria added. Once the 96-well microplate was loaded, it was placed in a TECAN plate reader which was used to measure absorbance at 600 nm to generate population growth curves. For bacterial samples the absorbance was measured every 15 min for a total of 8 h (32 cycles) in *S. aureus* and *P. aeruginosa* and for a total of 12.5 h (50 cycles) in *E. coli.* Preliminary trials indicated the number of cycles required for each species to reach *K* varied. In the fungal samples, absorbance was measured every 25 min (150 cycles) for approximately 63 h. All samples were maintained at 37 °C in the plate reader and underwent shaking to prevent clumping. Bacterial samples were subjected to continuous linear shaking at an amplitude of 5 mm while fungal samples were subjected to continuous shaking that alternated between orbital and linear shaking at an amplitude of 5 mm.

### Data analysis

The growth data generated by the TECAN plate reader was used to obtain estimates of *r* and *K* for each well of each 96-well plate using the package Growthcurver in Program R [19]. In the context of this experiment, estimates of *K* describe the maximum cell density in 202 μL of fluid, and reflects the population size at which rates of cell division are equal to rates of cell death. Estimates of *r* describe the maximum rate of increase in cell density during the exponential growth phase, and reflects the difference between the increased rate of cell division relative to rates of cell death. Estimates of *K* and *r* were then subjected to one-factor ANOVA with arborvitae dose treated as the main fixed effect to test whether there was a significant effect of arborvitae oil dose on the population parameters. In cases where ANOVA indicated a significant difference among arborvitae doses, post-hoc pairwise comparisons between doses were conducted using the lsmeans package which uses the Tukey method to control for multiple comparisons.

## Results

### Bacterial growth

#### *Escherichia coli*

**Fig. 1:**
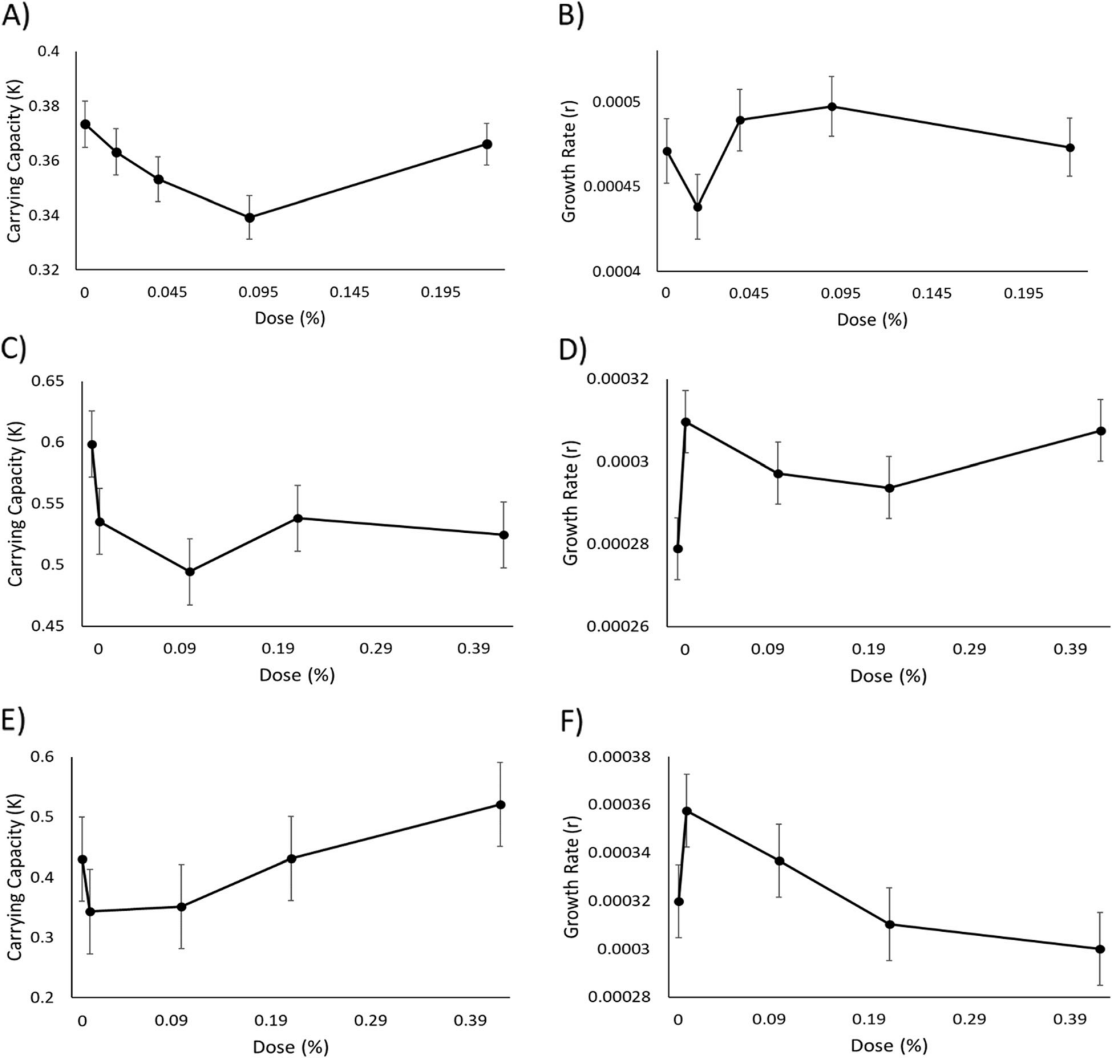
Estimates of the population parameters of bacterial species display the carrying capacity (left) in response to microdoses for *E. coli* (**a**), *S. aureus* (**c**), and *P. aeruginosa* (**e**) and the intrinsic rate of growth (right) in response to microdose for *E. coli* (**b**), *S. aureus* (**d**), and *P. aeruginosa* (**f**). Doses correspond to the percentage of arborvitae solution as indicated in Additional file 3: Table S1. Error bars are ±1 s.e.m.

One-way ANOVA indicated arborvitae oil significantly reduced *K* of *E. coli* at intermediate doses (df = 4; F = 2.6665 *p* = 0.04064; Additional file 3: Table S2, Fig. 1a). Specifically, post-hoc pairwise comparisons indicated a significant difference in *K* between doses of 0.000 and 0.090% (Additional file 3: Table S2). *r* for *E. coli* was not significantly impacted at any of the tested doses (df = 4; F = 1.5105; *p* = 0.2103; Fig. 1b; Additional file 3: Table S2).

One-way ANOVA indicated arborvitae oil significantly inhibited *K* of *E. coli* at the high concentrated dose and at the low concentrated dose (df = 3; F = 2144 *p* = 2.2 × 10^− 16^; Additional file 3: Table S2, Additional file 2: Figure S2 A). Specifically, post-hoc pairwise comparisons indicated significant differences in *K* and *r* between doses of 0.00 and 0.95% as well as 0.00% and .04% (Additional file 3: Table S2). *r* for *E. coli* was also significantly inhibited at both the high and low concentrated doses (df = 3; F = 271.83; *p* = < 2.20 × 10^− 16^; Additional file 2: Figure S2 A; Additional file 3: Table S2).

#### *Staphylococcus aureus*

One-way ANOVA indicated arborvitae oil ad no significant impact on *K* of *S. aureus* at any of the tested doses (df = 4; F = 1.9855; *p* = 0.1182; Fig. 1c; Additional file 3: Table S2). *r* for *S. aureus* significantly increased at low doses (df = 4; F = 2.7; *p* = 0.04633; Fig. 1d; Additional file 3: Table S2). Post-hoc pairwise comparisons indicated a significant difference in growth rate between doses of 0.000 and 0.008% (Additional file 3: Table S2).

One-way ANOVA indicated arborvitae oil significantly inhibited *K* of *S. aureus* at the high concentrated dose (df = 2; F = 22.282 *p* = 1.04 × 10^− 5^; Additional file 3: Table S2, Additional file 2: Figure S2 B). Specifically, post-hoc pairwise comparisons indicated a significant difference in *K* between doses of 0.00 and 0.95% (Additional file 3: Table S2). One-way ANOVA indicated marginally significant inhibitory effects of arborvitae oil on *r* (df = 2; F = 3.7325; *p* = .04293), but post-hoc pairwise tests with corrections for multiple comparisons showed there were no differences among pairs of the high or low concentrated doses.

#### *Pseudomonas aeruginosa*

One-way ANOVA indicated arborvitae oil had no significant impact on *K* (df = 4; F = 1.0668; *p* = 0.3876) or *r* (df = 4; F = 2.2555; *p* = .08288) for *P. aeruginosa* at any of the tested doses (Fig. 1e and f; Additional file 3: Table S2).

One-way ANOVA indicated arborvitae oil had no significant impact on *K* (df = 2; F = 0.7105; *p* = 0.504) or *r* (df = 2; F = 2.1996; *p* = .1383) for *P. aeruginosa* at either the high or low concentrated doses ( Additional file 3: Table S2, Additional file 2: Figure S2 C).

### Fungal growth

#### *Candida albicans*

**Fig. 2:**
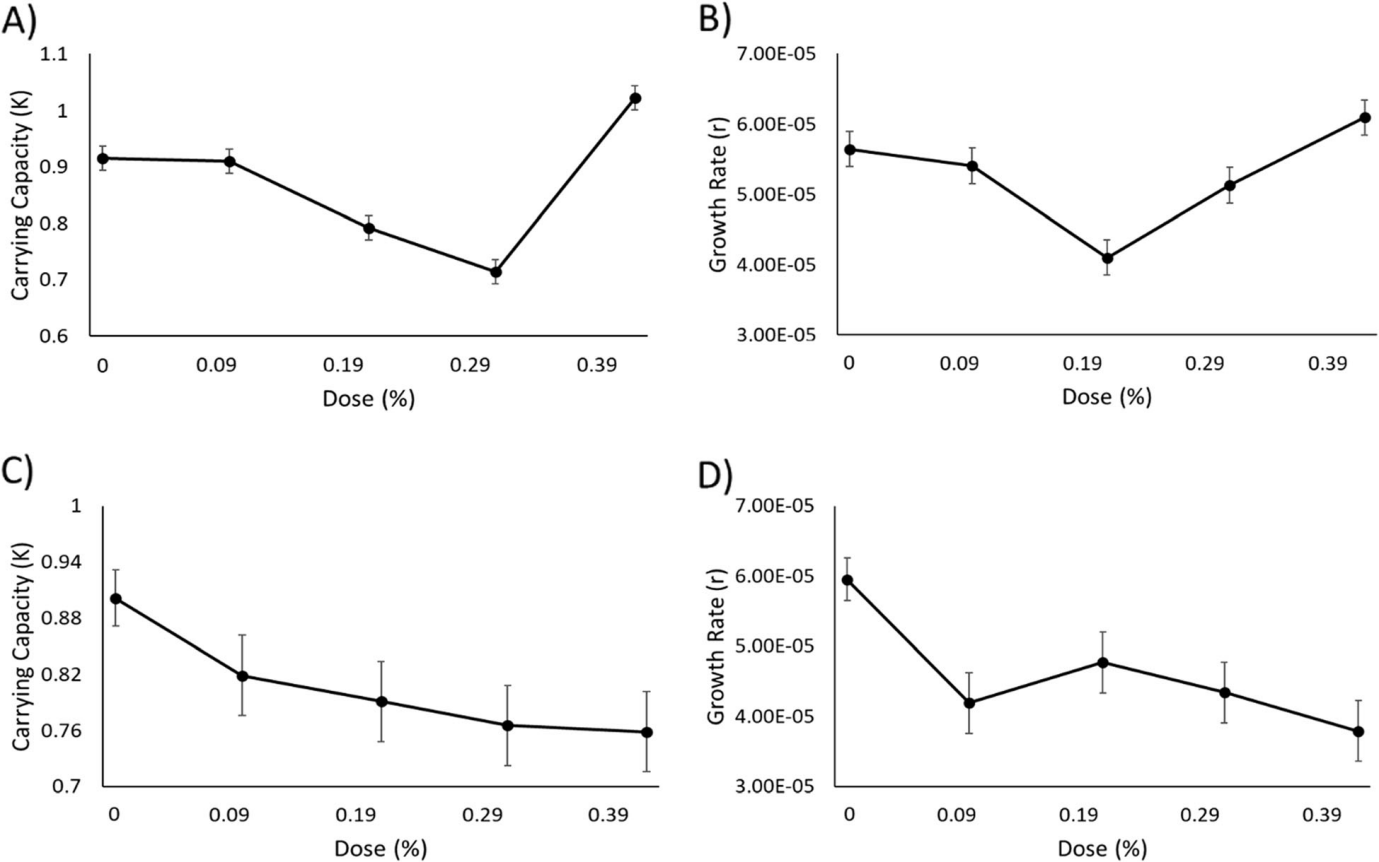
Estimates of the population parameters of fungi species display the carrying capacity (left) in response to microdoses for *C. albicans* (**a**) and *C. auris* (**c**) and the intrinsic rate of growth (r) in response to dose for *C. albicans* (**b**) and *C. auris* (**d**). Doses correspond to the percentage of arborvitae solution as indicated in Additional file 3: Table S1. Error bars are ±1 s.e.m.

One-way ANOVA indicated arborvitae oil significantly reduced both *K* (df = 4; F = 31.942; *p* = 3.07 × 10^− 11^) and *r* (df = 4; F = 8.7094; *p* = 5.47 × 10^− 5^) of *C. albicans* (Fig. 2a & b; Additional file 3: Table S2) at intermediate doses. Pairwise comparisons indicated there was a significant difference in *K* between all doses, except 0.00 and 0.10%, and 0.21 and 0.31% (Additional file 3: Table S2). The pairwise comparisons also showed there was a significant difference in *r* between the doses 0.21 and 0.00%, 0.21 and 0.10%, and 0.21 and 0.42% (Additional file 3: Table S2).

#### *Candida auris*

One-way ANOVA indicated marginally significant inhibitory effects of arborvitae oil on *K* (df = 4; F = 2.8325; *p* = 0.03592), but post-hoc pairwise tests with corrections for multiple comparisons showed there were no differences among pairs of doses (Fig. 2c; Additional file 3: Table S2). Differences between 0.31 and 0.00%, and 0.42 and 0.00% were close to significant based on pairwise comparisons using the Tukey method, and these most likely contributed to the marginally significant full-model ANOVA result. One-way ANOVA also indicated arborvitae oil inhibited *r* for *C. auris* (df = 4; F = 5.6725; *p* = 0.000931; Fig. 2d; Additional file 3: Table S2). The oil inhibited *r* of *C. auris* at low doses or at high doses, but not intermediate doses (Fig. 2d; Additional file 3: Table S2). Pairwise comparisons indicated a significant difference in *r* between 0.00 and 0.42%, 0.00 and 0.31%, and 0.00 and 0.10% (Additional file 3: Table S2).

## Discussion

This experiment was conducted to assess the ability of microdoses of arborvitae essential oil to alter the population growth parameters in both prokaryotic and eukaryotic microorganisms. Specifically, estimates of *K*, the carrying capacity, and *r*, the intrinsic rate of growth, were obtained to evaluate the potential use of arborvitae as an alternative to antimicrobials. In light of the observation that over two million individuals in the United States are infected with antibiotic resistant microbes, alternatives for combatting these microorganisms are necessary [1].

At least one active component of the essential oil arborvitae appears to be hydrophilic in nature. This determination is indicated by a significantly greater inhibition of *K* in *E. coli* with the hydrophilic solution compared to the untreated solution and hydrophobic solution (Additional file 2: Figure S1). Estimates of *r* did not differ between the hydrophilic and hydrophobic solutions, however, both were significantly reduced relative to the untreated solution (Additional file 2: Figure S1). The killing ability of the essential oil arborvitae was also evaluated in this study by comparing concentrated doses of the hydrophilic solution to microdoses of the hydrophilic solution. Results suggest that concentrated doses of arborvitae significantly inhibit both *K* and *r* in *E. coli* and *S. aureus,* but has no impact on *P. aeruginosa* (Additional file 2: Figure S2). The lack of effect of arborvitae on population parameters of *P. aeruginosa* could be due to unique membrane composition and protein function as *P. aeruginosa* has evolved mechanisms to deal with many antimicrobials. *E. coli* is of particular interest as the growth curve indicates rapid population decline and eventual extinction of the bacterial population. From these initial studies we were able to determine that at least one of the active components of arborvitae is hydrophilic, and that at concentrated doses is capable of completely inhibiting microbial growth of some species.

The primary goal of this experiment was to understand how microdoses of arborvitae effect the population parameters *K* and *r*, as essential oils are typically used in small concentrations. For example, diffusers are often used to disperse the essential oils into the air via water vapor. Essential oils are also frequently diluted for other uses such as oral rinses, household cleaning agents, and topical agents. Results suggest that the essential oil arborvitae, administered at microdoses, influences the population dynamics of microorganisms, including both Gram-positive and Gram-negative strains of bacteria and two fungal strains. Specifically, for the Gram-negative bacterium *E. coli*, *K* was significantly inhibited, specifically between the doses 0% and .09% (Fig. 1; Additional file 3: Table S2). However, *r* was not affected by the addition of arborvitae oil. The inhibition of *K* may be due to increased cell death that then equals the rate of cell division earlier in the exponential growth phase such that the population rates of cell division are equal to rates of cell death and therefore lower *K*. There was still a period of time in the exponential growth phase of *E. coli* where increased rates of cell division relative to rates of cell death, and consequently *r*, was not inhibited. Neither population parameter of a second Gram-negative bacterial species, *P. aeruginosa*, were significantly impacted by the presence of arborvitae oil at any of the tested doses. The lack of inhibition for *K* indicates there was no increase in cell death to equal the rate of cell division during the exponential growth phase, and therefore the population carrying capacity was not affected. *r* was also not inhibited, which could be due to the absence of increased cell death, or no decrease in cell division which also would not inhibit *r*. For *S. aureus*, *K* was not affected, but *r* was significantly increased, specifically between the doses 0% and .008%. The amplification of *r* may be due to the inability of these microdoses to increase cell death or decrease cell division, which ultimately magnifies the difference between the rate of cell division relative to rates of cell death. The absence of inhibition of *K* could be due to a lack of increase in cell death that counteracts the rate of cell division during the exponential growth phase, and therefore *K* was not affected.

In the fungal species *C. albicans,* both population parameters were significantly reduced at a variety of doses. Inhibition of *K* could be a result of increased cell death, allowing the rate of cell death to prematurely equal the rates of cell division during exponential growth, thereby lowering *K*. A lower maximum rate of increase in cell density during exponential growth phase could explain the observed decrease in *r*. The reduction in *K* of the second fungal species, *C. auris*, was marginally, significantly reduced by the essential oil arborvitae. However, there were no significant differences among the doses tested. Absence of significant inhibition of *K* could be due to a lack of substantial increase in cell death to equal the rate of cell division during the exponential growth phase, and therefore *K* was not affected. *r* for *C. auris* was significantly reduced at low or high doses of arborvitae, but not at intermediate doses. The higher and lower doses of the oil may have been able to lower *r* by increasing cell death or decreasing cell division, which may allow a smaller difference between the rate of cell division relative to rates of cell death. Overall, the essential oil arborvitae has the ability to inhibit one or more of the population parameters uniquely in all but one of the microorganisms (*P. aeruginosa*) tested.

Arborvitae oil had its most significant impacts on the population dynamics of fungal species, particularly *C. albicans*. These greater effects in fungal species, compared to any of the bacterial species, may be due to the structural differences between eukaryotic and prokaryotic cells. Specifically, a peptidoglycan cell wall is present in bacteria, and not in eukaryotic fungal cells. This cell wall may be a beneficial barrier that prevents the active components of the arborvitae oil from penetrating the cell, thus minimizing oil effects on the population dynamics of bacterial populations.

Within the bacteria, variability in the cell membrane and cell wall composition between Gram-positive and Gram-negative bacterial cells may also contribute to variation in susceptibility to arborvitae oil. We hypothesized that due to the thick peptidoglycan cell wall in Gram-positive bacteria (*S. aureus*) there would be a reduced effect of oil on the population parameters compared to the Gram-negative bacteria (*P. aeruginosa* and *E. coli*) which have thinner peptidoglycan cell walls. However, this hypothesis was not supported by these results as the population parameter *K* for the Gram-negative species *E. coli* was significantly impacted more than *K* for the Gram-positive species *S. aureus*. Additionally, the population dynamics of the second Gram-negative bacterial species, *P. aeruginosa*, was not altered in response to oil.

The differences in the ability of arborvitae oil to modify population dynamics of the Gram-negative strains of bacteria may also be explained by differences in membrane components, such as pumps and porins. Specifically, *P. aeruginosa* is frequently found in soils, and is likely tolerant of osmotic changes. *E. coli* can also be found in warm and temperate environments including contaminated soil, sediments, sands, algae, and the intestinal tracts of warm-blooded animals [20]. Thus, both species are Gram-negative and are often soil-dwelling. Consequently, they both have likely evolved mechanisms to tolerate osmotic stress through various forms of pumps, channels and osmolytes. However, *P. aeruginosa* has an outer membrane that is only 8% as permeable as the outer membrane of *E. coli* [21]. This reduced permeability may confer greater resistance to arborvitae oil in *P. aeruginosa* through one of two genetically controlled components: the outer membrane porin OprD and the multidrug efflux pump [22]. The OprD porins function to bring nutrients into the cell from the environment and are abundant in the membrane, but under stress these OprD porins are inhibited and no longer transcribed or translated in order to serve as a barrier which prevents drug entry into the cell [22]. The multidrug efflux pumps help to lower the accumulation of the drug in the cell via active export by membrane-associated pumps [22]. Additional studies have identified two channels, MscL and MsCS, that work together to release osmolytes and to maintain the membrane tension which allows membrane sealing and prevents the cell from crenating [23].

With the leading hypothesis for essential oil function involving penetration of the cell membrane by active components of the essential oils resulting in cell death [24], shuttling the water and drugs where needed to maintain membrane tension and cell function potentially allows bacterial cells to survive in the presence of essential oils such as arborvitae. In addition to population parameter variation among microbial species, there was also variability in population dynamics among doses within a species. One possible explanation for variation in population dynamics among doses is variation in the properties of the active component in arborvitae oil. Given the experimental procedure used to assess population dynamics it appears that arborvitae oil contains an active hydrophilic component that is able to inhibit population parameters in both fungal and bacterial species.

Continuation of this study will use mass spectrometry to identify the active component(s) of arborvitae oil that is/are responsible for population parameter inhibition and the overall antimicrobial properties of this essential oil. Based upon initial examination and experimentation, the active component will most likely be of a hydrophilic nature. Studies investigating the active components of other essential oils have also shown the active component to be volatile [25].

## Conclusions

The essential oil arborvitae significantly effects the population parameters across both bacterial and fungal species in a dose-dependent manner. It appears that the doses at which a microorganism is affected is species-specific. At concentrated doses, there is a strong negative impact on the carrying capacity, as this population parameter was significantly inhibited for both *S. aureus* and *E. coli.* In *E. coli*, there was complete cell death in response to these high doses. Although it is important that arborvitae essential oil can work at high doses, this experiment was focused on the impacts of microdoses on population parameters for bacterial and fungal species. *E. coli* was the most susceptible to microdoses of arborvitae. Interestingly, *S. aureus* had an increased intrinsic rate of growth in the presence of arborvitae with no significant effect on the carrying capacity. Likewise, *P. aeruginosa* also was not impacted. In general, the fungal species were more impacted than the bacterial species with both population parameters significantly inhibited for *C. albicans* and the intrinsic rate of growth significantly reduced for *C. auris*. Conclusions from this study show arborvitae has the ability to change the population parameters of some prokaryotic and eukaryotic microorganisms and may be a useful alternative to the current antibiotics.

## Additional files

Additional file 1: The raw data used for the analyses described in the manuscript. (XLSX 370 kb)

Additional file 2: **Figure S1.** Estimates of the population parameters of *E. coli* display the carrying capacity (left) and intrinsic rate of growth (right) in response to various environments including: Mueller-Hinton broth with no arborvitae oil added, arborvitae oil added directly to the Mueller-Hinton broth (hydrophobic), and arborvitae oil in a hydrophilic solution with the Mueller-Hinton broth. **Figure S2.** Growth curves were generated from the data produced by the TECAN for each of the bacterial species *E. coli* (top), *S. aureus* (middle), and *P. aeruginosa* (bottom) in the presence of MH broth only (solid line), a highly concentrated dose of .95% arborvitae oil/MH broth solution (dotted line), and a low concentrated dose of .04% arborvitae oil/MH broth solution (dashed line). These treatment solutions were made as shown in Additional file 3: Table S1 before being added to the appropriate wells in the plate for 18.75 h (75 cycles) with measuring absorbance at 600 nm every 15 min. The plate was also maintained at 37 °C and was shaken linearly for 900 s at amplitude 5 mm. (DOCX 379 kb)

Additional file 3: **Table S1.** Instructions for making the broth-oil solution and various assigned doses that were created and tested in this experiment. Broth indicated as MH in the table represents Mueller-Hinton broth for bacterial tests and GPP in the table indicates Glucose Phosphate Proline broth for fungal tests. Plate numbers correspond to which run and include the doses that correspond to the percentage of the solutions that were tested in each particular plate. A two-step dilution process was done to create the desired dose percentages. See methods section for a description of the percentages that corresponds to the doses that were created and used. **Table S2.** Results from ANOVA and t-tests. (XLSX 24 kb)

## Data Availability

All data generated for analyses during this study are included in this published article and its Additional file 1.

## Abbreviations

ANOVA: Analysis of variance
GPP: Glucose Phosphate Proline Broth
*K*: carrying capacity
MH: Mueller-Hinton Broth
*r*: intrinsic rate of growth
rpm: rotations per minute
