# Supplementary figures and images for: Microbial population dynamics under microdoses of the essential oil arborvitae
Source: BMC Complement Altern Med. 2019 Sep 5;19:247. doi: 10.1186/s12906-019-2666-6 (PMC6728999; doi:10.1186/s12906-019-2666-6)

**Figure S1:**


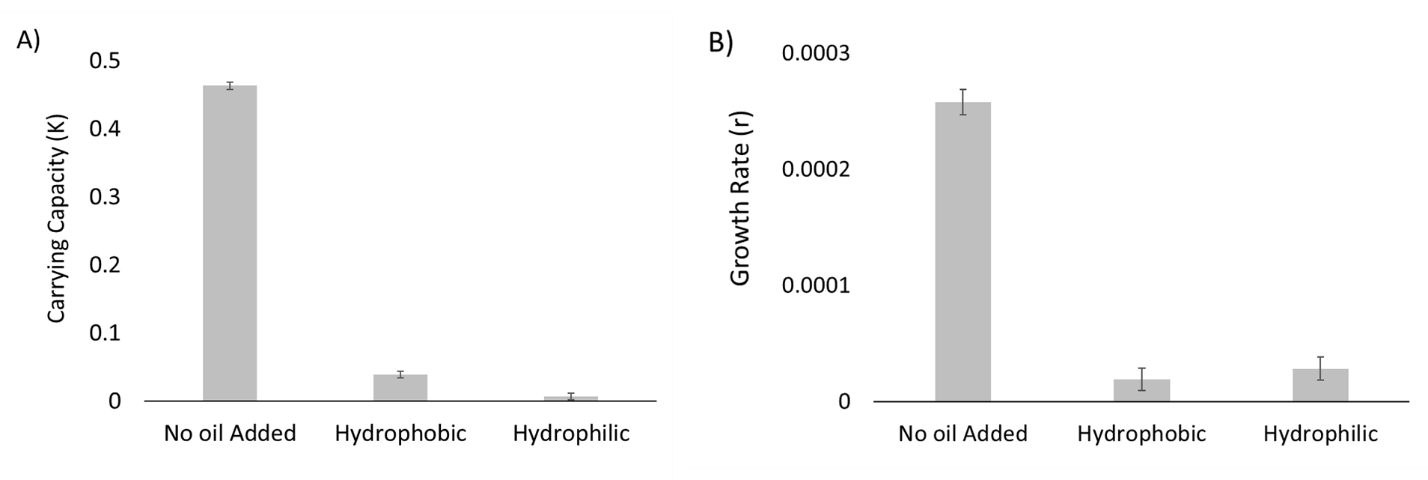


**Figure S2:**

**
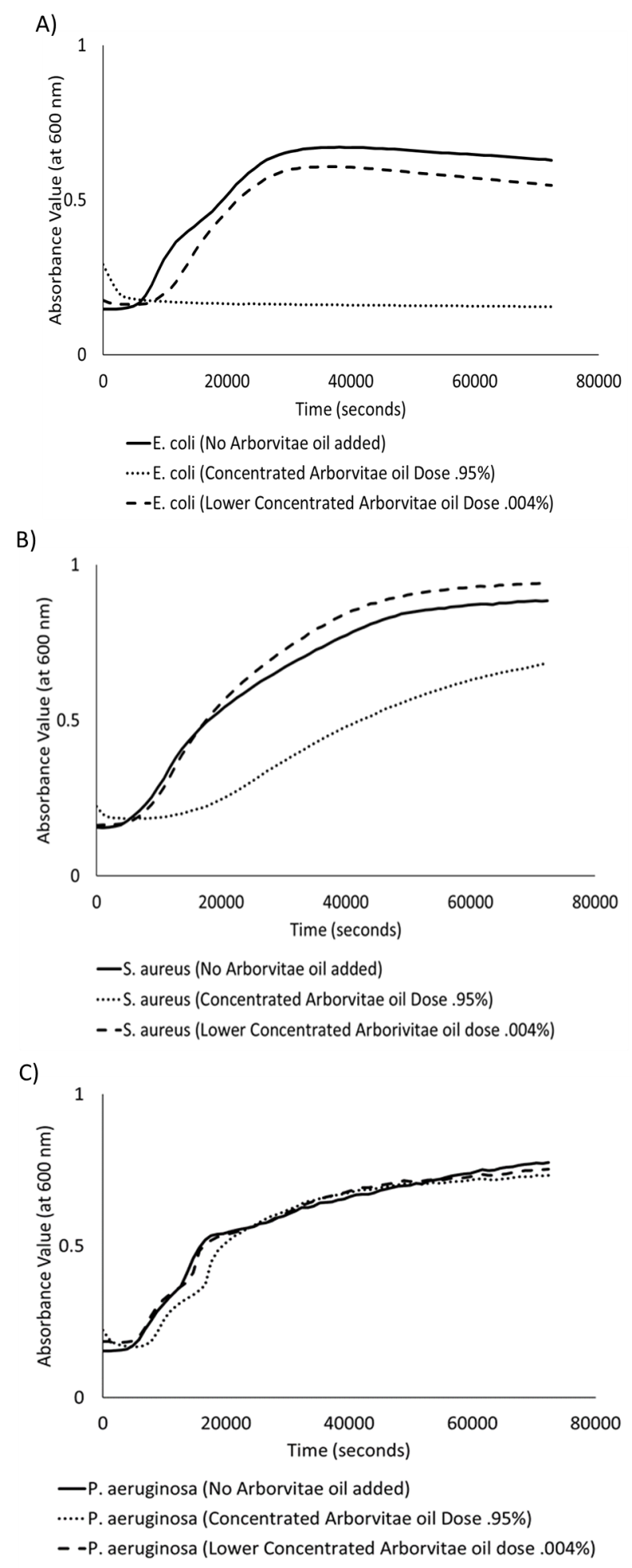
**

Supplement: Supplementary file 2 — Figure S1. Estimates of the population parameters of E. coli display the carrying capacity (left) and intrinsic rate of growth (right) in response to various environments including: Mueller-Hinton broth with no arborvitae oil added, arborvitae oil added directly to the Mueller-Hinton broth (hydrophobic), and arborvitae oil in a hydrophilic solution with the Mueller-Hinton broth. Figure S2. Growth curves were generated from the data produced by the TECAN for each of the bacterial species E. coli (top), S. aureus (middle), and P. aeruginosa (bottom) in the presence of MH broth only (solid line), a highly concentrated dose of .95% arborvitae oil/MH broth solution (dotted line), and a low concentrated dose of .04% arborvitae oil/MH broth solution (dashed line). These treatment solutions were made as shown in Additional file 3: Table S1 before being added to the appropriate wells in the plate for 18.75 h (75 cycles) with measuring absorbance at 600 nm every 15 min. The plate was also maintained at 37 °C and was shaken linearly for 900 s at amplitude 5 mm. (DOCX 379 kb) [file 12906_2019_2666_MOESM2_ESM.docx]
